# Complete genome sequence of *Ignisphaera aggregans* type strain (AQ1.S1^T^)

**DOI:** 10.4056/sigs.1072907

**Published:** 2010-08-20

**Authors:** Markus Göker, Brittany Held, Alla Lapidus, Matt Nolan, Stefan Spring, Montri Yasawong, Susan Lucas, Tijana Glavina Del Rio, Hope Tice, Jan-Fang Cheng, Lynne Goodwin, Roxanne Tapia, Sam Pitluck, Konstantinos Liolios, Natalia Ivanova, Konstantinos Mavromatis, Natalia Mikhailova, Amrita Pati, Amy Chen, Krishna Palaniappan, Evelyne Brambilla, Miriam Land, Loren Hauser, Yun-Juan Chang, Cynthia D. Jeffries, Thomas Brettin, John C. Detter, Cliff Han, Manfred Rohde, Johannes Sikorski, Tanja Woyke, James Bristow, Jonathan A. Eisen, Victor Markowitz, Philip Hugenholtz, Nikos C. Kyrpides, Hans-Peter Klenk

**Affiliations:** 1 DSMZ - German Collection of Microorganisms and Cell Cultures GmbH, Braunschweig, Germany; 2 DOE Joint Genome Institute, Walnut Creek, California, USA; 3 HZI – Helmholtz Centre for Infection Research, Braunschweig, Germany; 4 Los Alamos National Laboratory, Bioscience Division, Los Alamos, New Mexico, USA; 5 Biological Data Management and Technology Center, Lawrence Berkeley National Laboratory, Berkeley, California, USA; 6 Oak Ridge National Laboratory, Oak Ridge, Tennessee, USA; 7 University of California Davis Genome Center, Davis, California, USA

**Keywords:** hyperthermophile, obligately anaerobic, moderately acidophilic, fermentative, cocci-shaped, hot spring, *Crenarchaeota*, *Desulfurococcaceae*, GEBA

## Abstract

*Ignisphaera aggregans* Niederberger *et al.* 2006 is the type and sole species of genus *Ignisphaera*. This archaeal species is characterized by a coccoid-shape and is strictly anaerobic, moderately acidophilic, heterotrophic hyperthermophilic and fermentative. The type strain AQ1.S1^T^ was isolated from a near neutral, boiling spring in Kuirau Park, Rotorua, New Zealand. This is the first completed genome sequence of the genus *Ignisphaera* and the fifth genome (fourth type strain) sequence in the family *Desulfurococcaceae*. The 1,875,953 bp long genome with its 2,009 protein-coding and 52 RNA genes is a part of the *** G****enomic* *** E****ncyclopedia of* *** B****acteria and* *** A****rchaea * project.

## Introduction

Strain AQ1.S1^T^ (= DSM 17230 = JCM 13409) is the type strain of the species *Ignisphaera aggregans*, which is the type species of the genus *Ignisphaera* [1], one out of nine genera in the family *Desulfurococcaceae* [2-5]. The generic name derives from the Latin word ‘ignis’ meaning ‘fire’, and ‘sphaera’ meaning ‘ball’, referring to coccoid cells found in the high-temperature environment such as hot springs [1]. The species epithet is derived from the Latin word ‘aggregans’ meaning ‘aggregate forming or aggregating clumping’, referring to the appearance of the cells when grown on mono-, di- or polysaccharides [1]. Strain AQ1.S1^T^ is of particular interest because it is able to ferment quite a number of polysaccharides and complex proteinaceous substrates [1]. Here we present a summary classification and a set of features for *I. aggregans* AQ1.S1^T^, together with the description of the complete genomic sequencing and annotation.

## Classification and features

Strain AQ1.S1^T^ was isolated from a near neutral, boiling spring situated in Kuirau Park, Rotorua, New Zealand [1]. Interestingly, strains of *I. aggregans* could not be cultivated from pools with similar characteristics in Yellowstone National Park [1]. Only three cultivated strains are reported for the species *I. aggregans* in addition to AQ1.S1^T^, these are strains Tok37.S1, Tok10A.S1 and Tok1 [1]. The 16S rRNA sequence of AQ1.S1^T^ is 99% identical to Tok37.S1, 98% to Tok10A.S1 and 98% to Tok1. Sequence similarities between strain AQ1.S1^T^ and members of the family *Pyrodictiaceae* range from 93.0% for *Pyrodictium occultum* to 93.4% for *P. abyssi* [6] but from 89.7% for *Ignicoccus islandicus* to 93.5% for *Staphylothermus hellenicus* [6] with members of the family *Desulfurococcaceae* in which *I. aggregans* is currently classified (Table 1). Genbank [16] currently contains only three 16S rRNA gene sequences with significantly high identity values to strain AQ1.S1^T^: clone YNP_BP_A32 (96%, DQ243730) from hot springs of Yellowstone National Park, clone SSW_L4_A01 (95%, EU635921) from mud hot springs, Nevada, USA, and clone DDP-A02 (94%, AB462559) from a Japanese alkaline geothermal pool, which does not necessarily indicate the presence of *I. aggregans* but probably the presence of yet to be identified other species in the genus *Ignisphaera*. Environmental samples and metagenomic surveys featured in Genbank contain not a single sequence with >87% sequence identity (as of June 2010), indicating that *I. aggregans* might play a rather limited and regional role in the environment.

**Table 1:** Classification and general features of *I. aggregans* AQ1.S1^T^ according to the MIGS recommendations [7]

**MIGS ID**	**Property**	**Term**	**Evidence code**
	Current classification	Domain *Archaea*	TAS [8]
		Phylum *Crenarchaeota*	TAS [9,10]
		Class *Thermoprotei*	TAS [10,11]
		Order *Desulfurococcales*	TAS [10,12]
		Family *Desulfurococcaceae*	TAS [2-5]
		Genus *Ignisphaera*	TAS [1]
		Species *Ignisphaera aggregans*	TAS [1]
		Type strain AQ1.S1	TAS [1]
	Gram stain	not reported	
	Cell shape	regular or irregular cocci that occur singly, in pairs or in aggregates	TAS [1]
	Motility	none	NAS
	Sporulation	not reported	
	Temperature range	85°C–98°C	TAS [1]
	Optimum temperature	92-95°C	TAS [1]
	Salinity	< 0.5% NaCl	TAS [1]
MIGS-22	Oxygen requirement	obligate anaerobic	TAS [1]
	Carbon source	starch, trypticase, peptone, lactose, glucose, konjac glucomannan, amongst others (see text)	TAS [1]
	Energy source	carbohydrates, amino acids	TAS [1]
MIGS-6	Habitat	boiling spring	TAS [1]
MIGS-15	Biotic relationship	free-living	TAS [1]
MIGS-14	Pathogenicity	none	TAS [13]
	Biosafety level	1	TAS [13]
	Isolation	pool water	TAS [14]
MIGS-4	Geographic location	Kuirau Park, Rotorau, New Zealand	TAS [1]
MIGS-5	Sample collection time	2002	TAS [1]
MIGS-4.1	Latitude	176.24	TAS [14]
MIGS-4.2	Longitude	-38.13	TAS [14]
MIGS-4.3	Depth	≤ 2 m	TAS [14]
MIGS-4.4	Altitude	268 m	NAS

Evidence codes - IDA: Inferred from Direct Assay (first time in publication); TAS: Traceable Author Statement (i.e., a direct report exists in the literature); NAS: Non-traceable Author Statement (i.e., not directly observed for the living, isolated sample, but based on a generally accepted property for the species, or anecdotal evidence). These evidence codes are from of the Gene Ontology project [15]. If the evidence code is IDA, then the property was directly observed by one of the authors or an expert mentioned in the acknowledgements.

**Figure 1:**
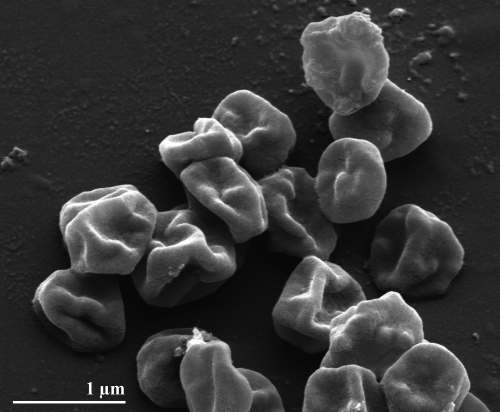
Scanning electron micrograph of *I. aggregans* AQ1.S1^T^

The cells of strain AQ1.S1^T^ are regular to irregular cocci which occur singly, in pairs or as aggregates of many cells [1]. They usually have dimensions between 1-1.5 μm (Figure 1). Aggregation of cells is common when AQ1.S1^T^ is grown on mono-, di- or polysaccharides [1]. Strain AQ1.S1^T^ is hyperthermophilic and grows optimally between 92°C and 95°C, the temperature range for growth is 85-98°C. The pH range for growth is 5.4-7.0, with an optimum at pH 6.4. The strain grows in the presence of up to 0.5% NaCl, however, it grows optimally without NaCl. The doubling time is 7.5 h under optimal conditions [1]. *I. aggregans* strain AQ1.S1^T^ is strictly anaerobic and grows heterotrophically on starch, trypticase peptone, lactose, glucose, konjac glucomannan, mannose, galactose, maltose, glycogen, and β-cyclodextrin. Growth on beef extract and glucose is weak and not observed on yeast extract, cellobiose, methanol, ethanol, trehalose, pyruvate, acetate, malate, casamino acids (0.1% w/v), carboxymethylcellulose, amylopectin (corn), xanthan gum, locust gum (bean), guar gum, dextran, xylan (oat spelts, larch or birch), xylitol, xylose or amylose (corn and potato) [1]. Mono- and disaccharides are accumulated in AQ1.S1^T^ cultures grown in media containing konjac glucomannan, but not in sterile media that had been exposed to the same temperature as the inoculated medium or the stock of konjac glucomannan [1].  As hypothesized by Niederberger *et al*. [1], this most probably indicates that the konjac glucomannan is being hydrolyzed enzymatically by AQ1.S1^T^ into sugars for metabolism. Removal of cystine from the growth medium does not affect cell density significantly. Hydrogen sulfide is also detected in AQ1.S1^T^ cultures grown in enrichment media. Strain AQ1.S1^T^ is resistant to novobiocin and streptomycin but sensitive to erythromycin, chloramphenicol and rifampicin [1].

### Chemotaxonomy

No chemotaxonomic data are currently available for *I. aggregans* strain AQ1.S1^T^. Also, chemotaxonomic information for the family *Desulfurococcaceae* is scarce. What is known is that the type species of this family, *Desulfurococcus mucosus*, lacks a murein cell wall and contains phytanol and polyisopreonoid dialcohols as major components of the cellular lipids [3].

**Figure 2:**
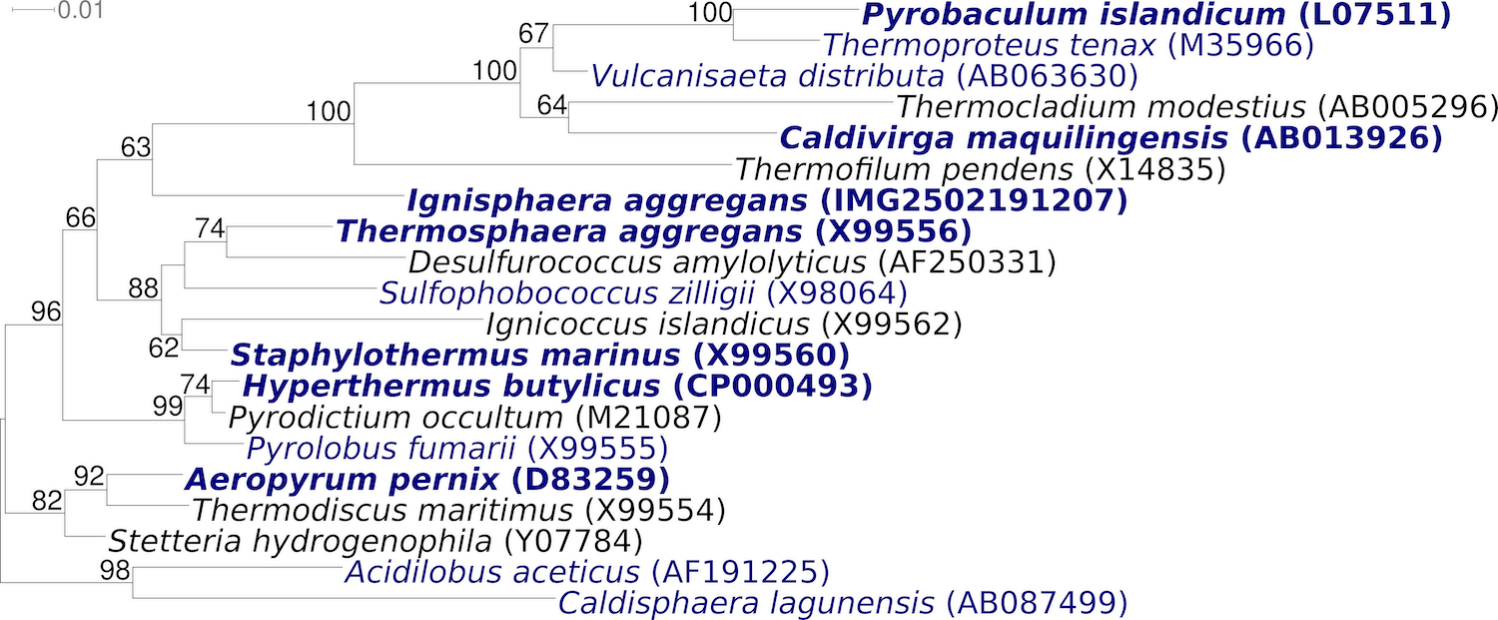
Phylogenetic tree highlighting the position of *I. aggregans* AQ1.S1^T^ relative to the type strains of the other genera within the order *Desulfurococcales*. The tree was inferred from 1,329 aligned characters [17,18] of the 16S rRNA gene sequence under the maximum likelihood criterion [19] and rooted with the type strains of the genera of the neighboring order *Acidilobales*. The branches are scaled in terms of the expected number of substitutions per site. Numbers above branches are support values from 250 bootstrap replicates [20] ,if greater than 60%. Lineages with type strain genome sequencing projects registered in GOLD [21] are shown in blue, published genomes in bold ([22-25], CP000504 and CP000852).

Figure 2 shows the phylogenetic neighborhood of *I. aggregans* AQ1.S1^T^ in a 16S rRNA based tree. The sequence of the single 16S rRNA gene copy in the genome of strain AQ1.S1 does not differ from the previously published 16S rRNA sequence from DSM 17230 (DQ060321).

## Genome sequencing and annotation

### Genome project history

This organism was selected for sequencing on the basis of its phylogenetic position [26], and is part of the *** G****enomic* *** E****ncyclopedia of* *** B****acteria and* *** A****rchaea * project [27]. The genome project is deposited in the Genome OnLine Database [21] and the complete genome sequence is deposited in GenBank. Sequencing, finishing and annotation were performed by the DOE Joint Genome Institute (JGI). A summary of the project information is shown in Table 2.

**Table 2:** Genome sequencing project information

**MIGS ID**	**Property**	**Term**
MIGS-31	Finishing quality	Finished
MIGS-28	Libraries used	Three genomic libraries: one 454 pyrosequence standard library, one 454 paired end 15 kb library, and one Illumina library
MIGS-29	Sequencing platforms	454 Titanium; Illumina GAii
MIGS-31.2	Sequencing coverage	94.5× pyrosequence and Illumina
MIGS-30	Assemblers	Newbler version 2.0.00.20- PostRelease-11-05-2008-gcc-3.4.6/, phrap, Velvet
MIGS-32	Gene calling method	Prodigal 1.4, GenePRIMP
	INSDC ID	CP002098
	Genbank Date of Release	August 24, 2010
	GOLD ID	Gc01330
	NCBI project ID	33361
	Database: IMG-GEBA	2502171146
MIGS-13	Source material identifier	DSM 17230
	Project relevance	Tree of Life, GEBA

### Growth conditions and DNA isolation

*I. aggregans* AQ1.S1^T^, DSM 17230, was grown anaerobically in DSMZ medium 1043 (*Ignisphaera* medium) [28] at 92°C. DNA was isolated from 0.5-1 g of cell paste using MasterPure Gram Positive DNA Purification Kit (Epicentre MGP04100). One µl lysozyme and five µl mutanolysin and lysostaphine, each, were added to the standard lysis solution for one hour at 37°C followed by 30 min incubation on ice after the MPC-step.

### Genome sequencing and assembly

The genome of strain AQ1.S1^T^ was sequenced using a combination of Illumina and 454 technologies. An Illumina GAii shotgun library with reads of 152 Mb, a 454 Titanium draft library with average read length of 320 bases, and a paired end 454 library with average insert size of 15 kb were generated for this genome. All general aspects of library construction and sequencing can be found at http://www.jgi.doe.gov/. Illumina sequencing data was assembled with VELVET and the consensus sequences were shredded into 1.5 kb overlapped fake reads and assembled together with the 454 data. Draft assemblies were based on 177 Mb 454 draft data, and 454 paired end data. Newbler parameters are -consed - a 50 -l 350 -g -m -ml 20. The initial assembly contained 20 contigs in 1 scaffold. The initial 454 assembly was converted into a phrap assembly by making fake reads from the consensus, collecting the read pairs in the 454 paired end library. The Phred/Phrap/Consed software package (http://www.phrap.com) was used for sequence assembly and quality assessment [29] in the following finishing process. After the shotgun stage, reads were assembled with parallel phrap (High Performance Software, LLC). Possible mis-assemblies were corrected with gapResolution (http://www.jgi.doe.gov), Dupfinisher [29], or sequencing cloned bridging PCR fragments with subcloning or transposon bombing (Epicentre Biotechnologies, Madison, WI). Gaps between contigs were closed by editing in Consed, by PCR and by Bubble PCR primer walks (J.-F. Chan, unpublished). A total of 32 additional reactions were necessary to close gaps and to raise the quality of the finished sequence. Illumina reads were also used to improve the final consensus quality using an in-house developed tool (the Polisher [30]). The error rate of the final genome sequence is less than 1 in 100,000

### Genome annotation

Genes were identified using Prodigal [31] as part of the Oak Ridge National Laboratory genome annotation pipeline, followed by a round of manual curation using the JGI GenePRIMP pipeline [32]. The predicted CDSs were translated and used to search the National Center for Biotechnology Information (NCBI) nonredundant database, UniProt, TIGRFam, Pfam, PRIAM, KEGG, COG, and InterPro databases. Additional gene prediction analysis and functional annotation was performed within the Integrated Microbial Genomes - Expert Review (IMG-ER) platform [33].

## Genome properties

**Figure 3:**
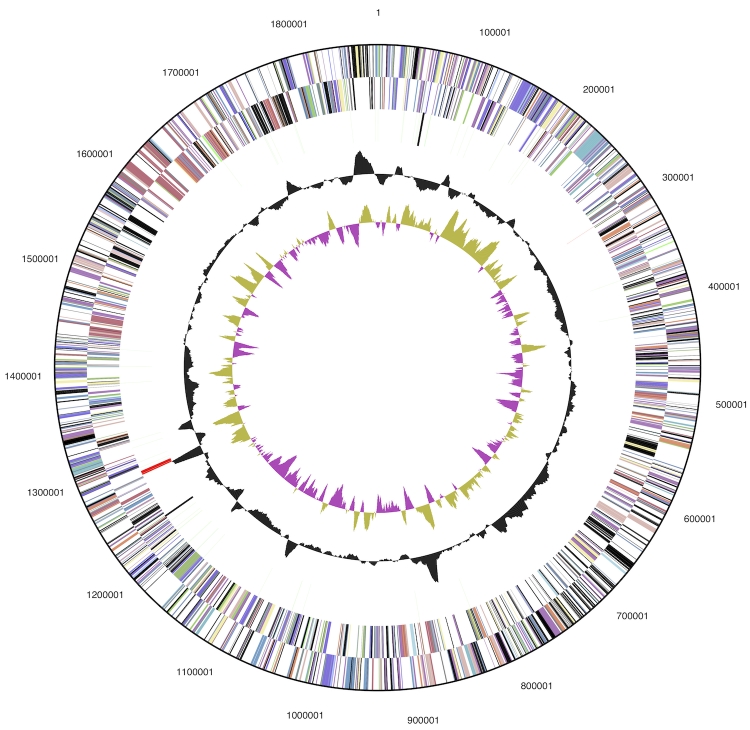
Graphical circular map of the genome. From outside to the center: Genes on forward strand (color by COG categories), Genes on reverse strand (color by COG categories), RNA genes (tRNAs green, rRNAs red, other RNAs black), GC content, GC skew.

The genome consists of a 1,875,953 bp long chromosome with a 35.7% G+C content (Table 3 and Figure 3). Of the 2,061 genes predicted, 2,009 were protein-coding genes, and 52 RNAs; 79 pseudogenes were also identified. The majority of the protein-coding genes (56.2%) were assigned a putative function while the remaining ones were annotated as hypothetical proteins. The distribution of genes into COGs functional categories is presented in Table 4.

**Table 3:** Genome Statistics

**Attribute**	**Value**	**% of Total**
Genome size (bp)	1,875,953	100.00%
DNA coding region (bp)	1,623,145	86.52%
DNA G+C content (bp)	669,463	35.69%
Number of replicons	1	
Extrachromosomal elements	0	
Total genes	2,061	100.00%
RNA genes	52	2.52%
rRNA operons	1	
Protein-coding genes	2,009	97.48%
Pseudo genes	79	3.83%
Genes with function prediction	1,158	56.19%
Genes in paralog clusters	190	9.22%
Genes assigned to COGs	1,220	59.19%
Genes assigned Pfam domains	1,280	62.11%
Genes with signal peptides	147	7.13%
Genes with transmembrane helices	454	22.03%
CRISPR repeats	9	

**Table 4:** Number of genes associated with the general COG functional categories

**Code**	**value**	**%age**	**Description**
J	158	11.9	Translation, ribosomal structure and biogenesis
A	2	0.2	RNA processing and modification
K	64	4.8	Transcription
L	70	5.3	Replication, recombination and repair
B	2	0.2	Chromatin structure and dynamics
D	9	0.7	Cell cycle control, cell division, chromosome partitioning
Y	0	0.0	Nuclear structure
V	22	1.7	Defense mechanisms
T	23	1.7	Signal transduction mechanisms
M	31	2.3	Cell wall/membrane/envelope biogenesis
N	11	0.8	Cell motility
Z	0	0.0	Cytoskeleton
W	0	0.0	Extracellular structures
U	16	1.2	Intracellular trafficking, secretion, and vesicular transport
O	56	4.2	Posttranslational modification, protein turnover, chaperones
C	82	6.2	Energy production and conversion
G	80	6.0	Carbohydrate transport and metabolism
E	135	10.2	Amino acid transport and metabolism
F	46	3.5	Nucleotide transport and metabolism
H	64	4.8	Coenzyme transport and metabolism
I	13	1.0	Lipid transport and metabolism
P	89	6.7	Inorganic ion transport and metabolism
Q	4	0.3	Secondary metabolites biosynthesis, transport and catabolism
R	209	15.8	General function prediction only
S	140	10.6	Function unknown
-	841	40.8	Not in COGs

## Insights from the genome sequence

**Figure 4:**
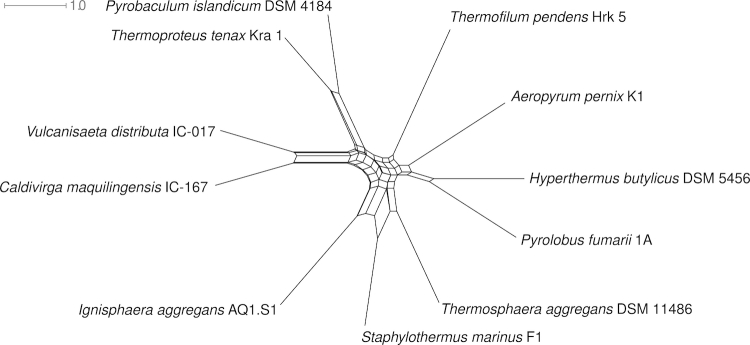
Phylogenetic network inferred from whole-genome (GBDP) distances, showing the relationships between *Desulfurococcaceae* (*Aeropyrum*, *Ignisphaera*, *Staphylothermus* and *Thermosphaera*), *Pyrodictiaceae* (*Hyperthermus* and *Pyrolobus*), *Thermoproteaceae* (*Caldivirga*, *Pyrobaculum*, *Thermoproteus* and *Vulcanisaeta*) and *Thermofilaceae* (*Thermofilum*).

Even though the tree depicted in Figure 1 is not particularly well resolved, the fact that *I. aggregans* does not cluster with the *Desulfurococcaceae* in 16S rRNA gene sequence-based phylogenies calls for a more detailed whole-genome-based analysis [34]. Both, in Figure 1 and in the All-Species-Living-Tree [35], *I. aggregans* is located deep on the branch leading to the *Thermoproteaceae* (and *Sulfolobaceae*). By circumstance, the class *Thermoprotei* within the phylum *Crenarchaeota* already offers a reasonably large set of reference genomes required for such an analysis. We thus assembled a dataset comprising all publicly available genomes from the set of organisms represented in the 16S rRNA tree (Fig. 1). Pairwise distances were calculated using the GBDP algorithm [36,37], which has recently been used to mimic DNA-DNA-hybridization values [37,38]. Here we applied the logarithmic version of formula (3) in [34,38]. The NeighborNet algorithm as implemented in SplitsTree version 4.10 [39] was used to infer a phylogenetic network from the distances, which is shown in Fig. 4.

The results indicate that the placement of *I. aggregans* as sister group of *Thermoproteales* (Fig. 1) is an artifact of the 16S rRNA analysis. The whole-genome network, while showing some conflicting signal close to the backbone, is in agreement with the splitting of the considered genera into the orders *Desulfurococcales* and *Thermoproteales*. However, the analysis provides some evidence that *Aeropyrum pernix* (*Desulfurococcaceae*) is more closely related to *Pyrodictiaceae* (represented by *Hyperthermus* and *Pyrolobus*) than to the remaining *Desulfurococcaceae*. The numerous additional type strain genome sequencing projects in the *Desulfurococcales* (Fig. 1) are likely to shed even more light on the phylogenetic relationships within this group by enabling future whole-genome phylogenies based on many more taxa.

A separate status of *I. aggregans* within the *Desulfurococcaceae* is supported by a lack of genes encoding membrane-bound multienzyme complexes that are thought to participate in the energy metabolism of members of this group. Operons encoding a MBX-related ferredoxin-NADPH oxidoreductase and a dehydrogenase-linked MBX complex are lacking in *I. aggregans*, although both are present in the completed genome sequences of *Thermosphaera aggregans* [24], *Staphylothermus marinus* [25] and *Desulfurococcus kamchatkensis*. The genome of *A. pernix* also lacks genes for the MBH-related energy-coupling hydrogenase, which are found in most members of the *Desulfurococcaceae* including *I. aggregans* (Igag_1902 – Igag_1914).
